# Sepsis may dissimulate a lymphoma? Case report

**DOI:** 10.1186/1471-2334-14-S7-P60

**Published:** 2014-10-15

**Authors:** Mihai Olariu, Valeriu Gheorghiță, Cristina Mihaela Olariu, Camelia Dobrea, Alina Elena Barbu, Adriana Nurciu, Florin Căruntu

**Affiliations:** 1 National Institute for Infectious Diseases "Prof. Dr. Matei Balş", Bucharest, Romania; 2 Carol Davila University of Medicine and Pharmacy, Bucharest, Romania; 3 Fundeni Clinical Institute, Bucharest, Romania; 4 University Emergency Hospital of Bucharest, Romania

## Background

In aggressive lymphoma, systemic “B” symptoms of fatigue, fever, night sweats may occur frequently, but these symptoms may occur also in severe sepsis. Many hematological disorders, especially lymphoid neoplasms, have a high risk for infection because of altered humoral and cell-mediated immunity.

## Case report

We present a case of a man 37 years old who was diagnosed in our clinic on July 2014 with diffuse large B-cell lymphoma. On July 5 the patient was admitted to the County Hospital Piteşti with fatigue, fever, night sweats and epigastric pain. Laboratory evaluation remarks a severe pancytopenia but without inflammation marks and a positive procalcitonin.

Marrow aspiration sample showed 28% lymphoid cells. The patient was transferred in our clinic with the diagnosis of sepsis. From family history we remark that his mother had died a few years back at 50 years old with acute leukemia. After clinical exam (pallor, splenomegaly), laboratory evaluation (severe pancytopenia: leukocytes 0.88x10^3^/µL, neutrophils 0.49x10^3^/µL, Hb 9.5 g/dL, platelets 25x10^3^/µL), LDH 1,704 IU/L, prothrombin time 16.5 s, fibrinogen 46 mg/dL, partial thromboplastin time 56.9 s, procalcitonin 3.95 ng/mL), abdominal CT scan, we considered intra-abdominal sepsis and disseminated intravascular coagulation (DIC).

Treatment for 7 days with meronem 3g/day, corticotherapy and supportive therapy, fresh frozen plasma, packed red blood cells, platelet concentrates did not ameliorate the clinical status and surgery exam disproved our suspicion. Because procalcitonin rose to 10.35 ng/mL treatment was completed with vancomycin 2g/day, caspofungin 50 mg/day, levofloxacin 1g/day, amikacin 1g/day and ethambutol 1g/day and also we performed a bone marrow biopsy.

After 5 days procalcitonin was 1.57 ng/mL and the clinical status was ameliorated but pancytopenia persisted. The histopathological exam and the immunohistochemistry aspect sustained the diagnosis of diffuse large B-cell lymphoma and the patient was transferred to the Hematology Clinic.

## Conclusion

In patients with severe infection who have a long evolution we must search for a hematological disease. Sometimes both hematological disease and sepsis may intertwine, being difficult to make a plain distinction.

## Consent

Written informed consent was obtained from the patient for publication of this Case report and any accompanying images. A copy of the written consent is available for review by the Editor of this journal.

